# Adapting Amidst Vulnerability: An Interpretative Phenomenological Analysis Study on Gay Men Living With HIV

**DOI:** 10.7759/cureus.58432

**Published:** 2024-04-16

**Authors:** Florian Thanasko, Maria Nikoloudi, Konstantina Antoniadou Anemi, Efi Parpa, Vassilis Kouloulias, Kyriaki Mystakidou

**Affiliations:** 1 Pain Relief and Palliative Care Unit, Department of Radiology, Aretaeio Hospital/National and Kapodistrian University of Athens School of Medicine, Athens, GRC; 2 Department of Developmental Psychology, National and Kapodistrian University of Athens School of Medicine, Athens, GRC; 3 2nd Radiology Department, Attikon Hospital/National and Kapodistrian University of Athens School of Medicine, Athens, GRC

**Keywords:** uncertainty, adaptation, vulnerability, gay men living with hiv, interpretative phenomenological analysis

## Abstract

Background: HIV represents a “biographical disruption”, interrupting the continuity of life and fostering a sense of vulnerability. The transition of HIV into a chronic condition, coupled with extended life expectancy, necessitates significant lifestyle adjustments, making adaptation and navigation through uncertainties essential.

Method: Interpretative phenomenological analysis was used to investigate the lived experiences and adaptation processes of gay men in Greece who are living with HIV. Semi-structured interviews were conducted with seven HIV-positive gay men, recruited from two Greek NGOs that support individuals living with HIV. Investigator triangulation was used to interpret textual material, heightening credibility and reducing bias, thereby enhancing the findings’ reliability.

Results: The analysis identified a superordinate theme, “Being Vulnerable Enough: Negotiating Uncertainties and Adapting in the HIV Experience”, which encompasses three themes: “The Moment of Division: Fear, Uncertainty, and Vulnerability after an HIV Diagnosis”, “Grief and Negotiation: Navigating Daily Life Through the Lens of Loss”, and “Reclaiming Self: Shaping ‘My HIV Identity’ to Fit on My Terms”.

Conclusions: The initial shock of HIV diagnosis introduces a sense of vulnerability, with participants confronting fear, despair, and grief over the loss of health and the disruption of their anticipated life flow. Being vulnerable enough enables individuals to adapt to life with HIV by managing uncertainties through creating certainties with small daily decisions, in a non-linear, ongoing process of negotiation and reassessment, without the need to eliminate all uncertainties.

## Introduction

More than three decades have passed since HIV was recognized as a chronic illness [1]. The advent of combined antiretroviral therapy has fundamentally shifted the perspective on HIV, turning what was once considered an inevitably fatal condition into a manageable chronic illness and extended life expectancy [2]. This development has led to the emergence of a wide breadth of theoretical models aimed at enhancing our comprehension of the chronic illness experience. These models include concepts such as illness identity [3], concealable stigmatized identities [4], and the HIV Stigma Framework [5].

Living with a chronic illness often necessitates significant lifestyle adjustments, presenting challenges that vary from person to person. While many patients successfully adapt, others may struggle, facing considerable effects on both their physical and psychological health [6].

However, it is important to acknowledge how various conditions uniquely affect individuals’ lives [7]. The modes of transmission for HIV, including unprotected sex or needle sharing, contribute to the stigma faced by people living with HIV (PLHIV), distinguishing it from other chronic diseases [8].

HIV represents a turning point in an individual's life narrative, described as a “biographical disruption” [9]. This disruption signifies a break in the continuity of one's life narrative, triggering rapid and extensive changes in various aspects of life [9-11]. The complex and slow progression of HIV to AIDS introduces significant uncertainty regarding the progression and alteration of symptoms, impacting individuals living with HIV [12].

Adapting to life after receiving an HIV-positive diagnosis involves redefining the meaning of HIV, setting a hopeful perspective, gaining control over life, seeking information, receiving support, and enhancing decision-making and communication skills [13-15].

Perrett and Biley created the Perrett and Biley Negotiating Uncertainty Theory posits that adapting to the uncertainties of an HIV diagnosis involves establishing new certainties amidst ongoing uncertainties [16]. They identified a series of adaptive steps for PLHIV, including considering risks, strengthening through hope, assuming direction, seizing opportunities, broadening vision, planning for the future, and maintaining life as it was prior to the diagnosis by finding ways that allow for continuity without alterations necessitated by the diagnosis.

In Greece, the adaptation processes and uncertainty for PLHIV might be influenced by persistent HIV-related stigma and decreased social support [17,18], affecting participants’ willingness and ability to engage in network interventions and potentially leading to significant limitations and discrimination [19]. These challenges are intensified by the country's recent severe financial crisis, which has negatively impacted the lives of PLHIV and their access to healthcare services [17].

Understanding a person's illness experience is essential for providing effective and compassionate care. Our qualitative study aims to understand the lived experiences of gay men living with HIV (GMLHIV) in Greece, exploring their adaptation processes after receiving an HIV-positive diagnosis.

## Materials and methods

Participants, recruitment, and ethics

In January 2020, the authors coordinated with managers from nationwide Greek NGOs supporting individuals living with HIV, who then reached out to potential participants. This led to seven homosexual male participants enrolling in the study. They received information about the study's objectives and provided written consent. Demographic details are presented in Table 1. The study was approved under the license number 226/18-06-2020.

**Table 1:** Sample’s demographic characteristics

Pseudonym	Age (years)	Living conditions	Employment	Living with HIV (years)
Sophocles	39	Alone	Full time	9
Hermes	43	Alone	Full time	2.5
Zois	41	Parental family	Full time	11
Procopius	33	With partner	Full time	7
Alexander	32	Alone	Part time	2.5
Kostas	29	Roommate	Part time	4
Peter	33	Alone	Part time	2.5

Data collection

From January to February 2020, seven semi-structured interviews, each lasting about 50 minutes, were conducted with participants. These interviews were audio-recorded, transcribed verbatim, and translated into English. Participants also filled out a demographic questionnaire covering age, living conditions, employment, and years living with HIV. The study's purpose was clearly explained to participants, ensuring their understanding. To maintain anonymity, all names mentioned in the transcripts are pseudonyms. The main question was: “What does living with HIV mean to you?”

Data analysis

We used interpretative phenomenological analysis (IPA) for data analysis, a qualitative approach aimed at understanding how individuals perceive their significant lived experiences [20,21]. IPA involves three main components: phenomenology, focusing on experiential knowledge and the personal lifeworld; hermeneutics, emphasizing the interpretation of deeper meanings beyond the obvious; and idiography, highlighting the uniqueness of individual experiences [20,21]. Phenomenology explores subjective perspectives rather than objective realities, requiring interpretation of experiential material. Hermeneutics encourages exploring underlying content, while idiography stresses the importance of context-specific, detailed analysis to understand how phenomena are uniquely perceived. The IPA methodology values the use of metaphors for participants to articulate and reflect on their experiences, enabling vivid descriptions that capture life's intricate nuances across multiple sensory dimensions [22].

IPA involves six steps: 1) Immersing in the data through repeated reading, focusing on the participant; 2) Taking initial notes and analyzing semantic content; 3) Developing emergent themes incorporating both participant's words and analyst's interpretations; 4) Identifying connections among themes; 5) Repeating the process for subsequent cases; and 6) Seeking patterns across cases [20,21].

Investigator triangulation

This study employed investigator triangulation, with the first three authors independently analyzing the data before collaborating. This approach aimed to enhance the findings' credibility and validity by minimizing bias and enriching interpretations with diverse perspectives [23]. Such collaboration ensured a coherent and plausible interpretation, bolstering the reliability and depth of understanding of the studied phenomenon [23].

## Results

From the data interpretation, life with HIV has been described as a biographical disruption bisecting life into pre- and post-diagnosis phases. Based on the analysis, the superordinate theme identified is “Being Vulnerable Enough: Negotiating Uncertainties and Adapting in the HIV Experience”, which is further divided into three themes (Table 2).

**Table 2:** Superordinate theme and the three themes

Superordinate theme: Being Vulnerable Enough: Negotiating Uncertainties and Adapting to the HIV Experience
The Moment of Division: Fear, Uncertainty, and Vulnerability after an HIV Diagnosis Grief and Negotiation: Navigating Daily Life Through the Lens of Loss Reclaiming Self: Shaping “My HIV Identity” to Fit on My Terms

Theme 1. The moment of division: fear, uncertainty, and vulnerability after an HIV diagnosis

The HIV diagnosis evoked the emotion of “fear”, accompanied by physical sensations of “numbness”, a feeling of “turning grey” and a sense of “cold”. For some, the diagnosis shock was more intense described as “despair”, “an explosion” and “fainting”. Participants expressed uncertainties regarding the potential shortening of life expectancy.

Sophocles: Grief, fear, despair. HIV gives you a kick in the ass and says, do you know that saying, “you only live once, and this is it”, making you understand it deeply and a bit more fearfully. You have questions about your life and how much longer you will live.

Alexander: Everything felt as if I were on a death row.

The uncertainty of an HIV-infected future casts a shadow over life's fragility, prompting reflections on life's brevity and disruption of the anticipated flow of existence.

Hermes: I was afraid of the side effects of the drugs, I feared my body would change, would I become depressed?

Alexander: It was like anticipating my own death (laughs), because I was also very uninformed about how we deal with the virus today.

The confrontation with mortality and existential questions, coupled with the inability to foresee the consequences of illness or treatment, intensify the feelings of vulnerability along the disease trajectory.

Theme 2. Grief and negotiation: navigating daily life through the lens of loss

Transitioning from the initial shock of diagnosis, participants face a period of isolation and absence from sex, burdened with the “responsibility”, “fear”, and “guilt” of not transmitting the virus to others. This pursuit of “loneliness”, indicative of the behavioral aspect of grieving, becomes a pivotal factor, as it challenges existing social roles and affects engagement in social networks, marking a significant disruption in one’s social fabric. Envisioning future relationships feels akin to evading them, amidst the uncertainty as a participant quotes “Who will want me now like this, broken, damaged?”.

Hermes: I communicate and flirt with people from abroad, because I know there's no chance I'll meet them. In other words, I do it from my own comfort zone.

Following the HIV diagnosis, participants begin to comprehend the magnitude of what has been lost. This realization of health loss precipitates disturbances in their identities and disintegrates other roles they once held.

Hermes: I am not who I used to be, it is as if I am missing a piece, that I feel it was taken from me by force, my health. I lost my health.

Hermes: I do not have a good self-image and for a very long time, I saw that who I was, all the things I have done in my life had become very small and my seropositivity had become a huge thing. It took all available space and had left everything else I had done in my life out and made them really-really small.

These metaphors unveil an unwelcome entity within, an internal reality. HIV exists inside.

Peter: It is something that exists within me. It is my child. It is an “it”, no male or female. It is my child. Interviewer: What does it look like? Peter: it looks like me, but not the present me. With me when I was a young carefree child … I don’t like it, I will be honest, I don’t like it.

Although the focus has shifted away from the diagnosis itself, the perspective has not expanded but instead remains self-centered, interpreting the new self through the lens of the diagnosis, as if HIV is destroying life.

Hermes: I am a time bomb ready to explode.

Alexander: Back then, I used to say that my life was ruined. Like a scattered puzzle. A puzzle with its pieces crumbled. An explosion. Like someone had a puzzle and dropped it on the floor.

Both life and self-image appear to be under the control of HIV. However, gradually, “you learn to live with it”. Managing daily life was challenging for many; for some, it took months, while for others even more. Reestablishing control was triggered by “good test results”. Nearly all participants regarded the combination therapy and its positive outcomes as a means of gaining control over the virus, prompting them to seek additional information from their physicians.

Sophocles: The bonuses of HIV, which come with free exams two or three times a year, with a doctor who knows you inside out. I'm not troubled, we don't run around from here to there, we don't worry.

Sophocles: When I get good test results. I am very happy, very optimistic, and you feel very strong.

Participants are mitigating vulnerability by establishing certainties in their daily lives, fostering optimism, balance, and the emergence of hope. This is because HIV has transitioned to “It's life now, the everyday reality”. “It has become part of my daily routine, and I have found a bit of my balance again”. Another method of gaining control involved changing their diet, regular testing for other sexually transmitted diseases, and adopting healthier daily routines. These seemingly minor adjustments in lifestyle help to slightly reduce the impact of uncertainties.

Theme 3. Reclaiming self: shaping “my HIV identity” to fit on my terms

Participants are accepting “my HIV identity” as a component of their self-identity, rather than the dominant aspect. This acceptance is creating room for the resurgence of roles and interests that were previously sidelined due to the diagnosis, fostering self-love.

Kostas: I was trying to re-identify myself; to adapt myself to the new situation.

Hermes: Seropositivity, at this moment, has taken a much smaller place in my life HIV is a trait, an element of my character, but that it's not me, I am also that. For a time, I was thinking that I am just that (seropositive), oh by the way, I am also a good friend, a good lover, and a good uncle. 

One pathway that facilitated this transition was the reevaluation of life's priorities, moving from prioritizing the care for others, and seeking the acceptance of others to placing themselves at the forefront of their own care and concerns.

Hermes: Before I did things for others.

Procopius: I was trying to please everyone. I didn't love myself at all and I did not protect it. If you don't love yourself, you will suffer. I please others, I don't live my own life, the things I want.

Alexander: I oppressed myself in order to be liked by certain people. It was as if I did not allow myself to be disliked by others until the diagnosis was made.

In essence, this transition marked a shift from self-neglect and the suppression of personal desires and identity aspects to conform to societal norms or sustain relationships, towards recognizing some positive aspects of living with HIV.

Sophocles: It (HIV) leads you to make yourself a priority; HIV is a bit selfish.

Hermes: I became more self-efficient; in a way that I am not easily influenced by others. I don’t do things that I don’t like anymore. 

Alexander: I can now say that I do not want to deal with you right now without remorse or the need to be polite. I started to put boundaries.

Some participants describe the shift from pre-HIV life to living with HIV as a transition toward adulthood and maturity. This was displayed through practical actions like leaving their parental home to live independently, and with improved ability to listen to others more attentively than before. However, accepting HIV as a part of oneself is challenging, and the process of adaptation is not straightforward. The sentiment “HIV is a bit selfish” encapsulates the internal conflict experienced.

Alexander: I decided that now I'm protecting myself, something like a self-preservation instinct.

Procopius: HIV means a major life lesson, a redefinition of life. Interviewer: what title would you give to this life lesson? Procopius: To love yourself more. I did not love myself at all and I did not protect him.

Shifting focus from oneself to others and then back to oneself can be particularly challenging, as it involves a constant negotiation of attention and care. This dynamic reflects a need for self-protection while also expanding one's focus beyond oneself, a delicate balance between vulnerability and ownership of one's HIV status.

The narratives on considering risks in sexual behavior evolve from initial denial to negotiating risk, and ultimately, for some, to acceptance.

Procopius: I essentially got infected because he wasn't taking his medication … Seropositivity as it happened, it was a moment caught in the heat of passion.

Sophocles: Maybe I engaged in a bit more unprotected sex than usual … We blame the person who infected us, ignoring the fact that when we got infected, we were also there. Who infected me? I went and got infected.

Peter: It happened in a dirty way, I was deceived … If he had informed me that he was diagnosed with it, and would like to be together, I might have accepted it … I got infected!

Interviews showed that before becoming infected, individuals harbored a deep fear of HIV. However, a transformative shift in perception occurred after diagnosis, wherein living with HIV actually diminished their fears, offering a sense of relief and freedom.

Sophocles: And one of the good things about HIV is that with the diagnosis you automatically stop being afraid that you will get HIV and only when you are diagnosed with HIV and you experience it, you realize how torturous this fear was. I had the fear of getting HIV all the time, every time I had sex that fear was present. I just couldn't understand how life without that fear was, when I realized the loss of this fear I finally understood what had happened in my life: HIV made me feel carefree and helped me get rid of the fear of HIV, it ceased to exist.

Alexander: I overcame a great fear. I even avoided testing. I was very afraid of the possibility of getting HIV. Why do I consider HIV as a supportive element? That is, there are times when I face things that I fear, just the memory of HIV (laughs) how much worse can everything else could be? This gives me courage, it's kind of like I have discovered (laughs) something that others have not discovered for themselves. Somewhat like a little secret, it helps me feel better. Released like a river. Liberated.

Peter: I had heard that I might have lung cancer, then they said I didn't, so I must have that (HIV). Thus, the big thing became small, making acceptance easier.

Zois: They couldn't find what I had, they were talking about cancer, so when I was finally diagnosed as HIV positive, it was more of a relief.

The transition from the initial fear of HIV infection to empowerment after diagnosis offers a cathartic release. Paradoxically, becoming infected with HIV relieves participants from the ongoing fear, turning the diagnosis into a form of support for a life free of fear, more optimistic and hopeful for the future. However, adapting to life with HIV is a non-linear journey, marked by ongoing uncertainties. The process involves continuous adjustments and coping with the unpredictability of living with the virus.

Procopius: I am fighting. Interviewer: Could you assign a form to what you are fighting with? Procopius: Form like fighting with evil so that it prevails over good. Good and Evil. Which will survive?

## Discussion

This study explored the experiences of GMLHIV in Greece, focusing on how they adapt following a positive diagnosis. While adaptation levels varied among participants, common pathways to achieving it were noted. The journey began with the diagnosis; a moment of division disrupting life's expected flow and causing emotional suffering. This led to uncertainties, including concerns about mortality and treatment side effects, heightening feelings of vulnerability in alignment with other studies [15,16,24,25]. Participants used personal metaphors to comprehend their illness, a method observed in studies of similar conditions [22,24,25]. These metaphors encompassed emotions, physical sensations, and appearance, linking the uncertainties of the virus to familiar experiences, in order to feel less vulnerable. 

Participants experienced grief realizing their losses, encapsulated in the sentiments “I am not who I used to be; it's as if I'm missing a piece” and “lost my carefree days”. This reflects a deep disturbance in identity and a cognitive disruption akin to grieving, similar to feelings of loss over a deceased loved one as described by Shuchter and Zisook [26]. However, in this context, the loss pertains to health and the carefree nature of life before the diagnosis, highlighting a different form of grief. Grieving for participants was a solitary process, marked by abstaining from sexual activity due to the responsibility and fear of transmitting the virus, impacting their well-being. This resonates with findings from previous studies, highlighting a common experience of isolation in the context of HIV [27]. While another study on gay men in Greece associated conscious loneliness with grieving in the face of mortality [15], our research expands more on that. It suggests that isolation not only facilitated a confrontation with mortality but also provided them the space and the opportunity to reflect on HIV's control in their lives. Participants began to regain control over their lives from the influence that HIV once had, through gradual, small decisions regarding daily routines, such as better nutrition, consistent testing for sexually transmitted infections, and positive feedback from HIV treatment.

Participants embraced HIV as part of their identity, asserting control over it as “My HIV Identity”. This contrasts with studies on heterosexual men, who often view HIV more as an unfortunate event rather than an integral aspect of their identity [28]. For the participants, integrating their HIV identity was a complex journey marked by the realization that “HIV is a bit selfish”. Through vulnerability, they transitioned from seeing HIV as life-destroying to managing, and in some cases, appreciating it as life-saving. Adaptation to living with HIV doesn't necessarily require eliminating all uncertainties. Creating certainties through small decisions can facilitate adaptation, which is inherently non-linear and involves a continuous process of negotiation and reassessment. Our findings reinforce Perret and Biley's negotiation uncertainty theory [16], notably within a cultural context where HIV stigma is prevalent [17,18]. Our study enhances the dialogue on hope by expanding its context beyond just anticipating a cure, as found in another research study [15]. Participants discovered a sense of relief upon receiving their HIV diagnosis, contrasting it with the dread of potentially more severe diagnoses. Furthermore, participants transitioned from fearing HIV contraction to embracing a life with HIV. This “maturity” reflects a journey from past fears and self-neglect, through making impactful daily decisions and finding empowerment in positive test results, to envisioning a more optimistic future.

Limitations of the study

Despite the small sample size of seven participants, it’s appropriate for an IPA study, which focuses on deep, detailed explorations of personal experiences. The data collected from our study was extensive and rich. The study explored the adaptation of GMLHIV and did not capture experiences of women and heterosexual men living with HIV or AIDS. The results presented cannot claim to represent how these populations may adapt to HIV, indicating a need for broader research to fully grasp the diverse adaptations to living with HIV.

## Conclusions

This study informs healthcare professionals, about the challenges GMLHIV face, emphasizing the importance of self-care and changes in self-perception. Healthcare providers should recognize that eliminating uncertainties isn’t always necessary for adaptation; it can arise by creating certainties amidst uncertainties and vulnerability. 

Clinical considerations for healthcare professionals involve encouraging patients to seek information on their condition, take control of decision-making regarding daily life decisions, and engage in self-reflection across the past, present, and future. This approach can help patients to adapt life with HIV in a more optimistic way.
